# A multicenter retrospective study: risk factors and nomogram construction for pathological escalation of gastric low-grade intraepithelial neoplasia

**DOI:** 10.3389/fmed.2026.1800141

**Published:** 2026-04-30

**Authors:** Di-yun Shen, Shun-hai Zhou, Chao-yi Shi, Xuan-ran Chen, Yu-zhen Bi, Xue-man Wang, Yan Sun, Jun Zhang

**Affiliations:** 1 The First School of Clinical Medicine, Zhejiang Chinese Medical University, Hangzhou, Zhejiang, China; 2 The Second Clinical Medical College, Zhejiang Chinese Medical University, Hangzhou, Zhejiang, China; 3 The Second School of Clinical Medicine, Hangzhou Normal University Hangzhou, Zhejiang, China; 4 Department of Gastroenterology, Chun’an County First People’s Hospital (Zhejiang Provincial People’s Hospital, Chun’an branch), Hangzhou, Zhejiang, China; 5 Endoscopy Center, Shaoxing People’s Hospital, Shaoxing, Zhejiang, China; 6 Department of Gastroenterology, Zhejiang Provincial People’s Hospital (Affiliated People’s Hospital, Hangzhou Medical College), Hangzhou, Zhejiang, China; 7 Department of Gastroenterology, The First Affiliated Hospital of Zhejiang Chinese Medical University (Zhejiang Provincial Hospital of Chinese Medicine), Hangzhou, Zhejiang, China

**Keywords:** clinical validation, intraepithelial neoplasia, multicenter, nomogram, pathological escalation

## Abstract

**Background:**

Low-grade intraepithelial neoplasia (LGIN) is recognized as a precancerous lesion of gastric carcinoma. However, discrepancies have been observed between initial biopsy findings and post-Endoscopic Submucosal Dissection (ESD) histopathological results. This study aims to develop and clinically validate a multifactorial nomogram for predicting the risk of pathological upgrading in gastric LGIN.

**Methods:**

We retrospectively analyzed clinical data from patients who underwent gastric ESD at three hospitals from June 2018 to July 2024. Sixty-eight patients from Zhejiang Provincial Hospital of Chinese Medicine and fifteen patients from Zhejiang Provincial People’s Hospital constituted the training cohort; sixty-eight patients from Shaoxing People’s Hospital formed the validation cohort. We performed detailed comparisons of 13 baseline patient characteristics and conducted logistic regression analyses. Subsequently, a nomogram was developed and evaluated.

**Results:**

The pathological upgrade rate was 54.1%. Independent risk factors included male sex, mixed IIc lesion morphology, clear demarcation lines, lesion size ≥ 15 mm, and Helicobacter pylori antibody positivity. The area under the receiver operating characteristic curve (AUC) of the training cohort was 0.891, with a sensitivity of 0.882 and specificity of 0.592, indicating strong discriminative ability. The calibration curve closely aligned with the diagonal, suggesting excellent predictive accuracy. DCA indicated significant clinical benefit. The predictive performance of the constructed nomogram in the validation cohort is moderate, with an AUC of approximately 0.67.

**Conclusion:**

This multicenter retrospective study identified several independent risk factors for pathological escalation of LGIN to high-grade intraepithelial neoplasia (HGIN) or gastric cancer, including mixed lesion morphology, clear demarcation lines, and lesion size ≥ 15 mm. Further prospective studies are warranted to validate the nomogram’s performance across diverse clinical settings.

## Background

In recent decades, advances in medical technology and health awareness have improved gastric cancer prevention and control. Standardized screening guidelines (1) and improvements in living conditions, diet, and Helicobacter pylori (Hp) eradication (2) have contributed to declining incidence (3). At the same time, more and more studies have begun to look at precancerous lesions such as mucosal atrophy, intestinal metaplasia, and dysplasia (4). These precancerous lesions are considered to be an important intermediate stage in the development of stomach cancer, therefore, early detection and treatment of stomach cancer and its precancerous lesions is essential to improve the survival rate and quality of life of patients (5).

Gastric dysplasia is an important precancerous lesion, also known as intraepithelial neoplasia or non-invasive neoplasia (6). Based on the degree of cellular atypia and lesion severity, it is divided into low-grade intraepithelial neoplasia (LGIN) and high-grade intraepithelial neoplasia (HGIN). The classification is directly related to the risk of gastric cancer. The 2019 World Health Organization (WHO) Classification of Tumors of the Digestive System (7) defined LGIN as a definite epithelial neoplastic proliferation with mild to moderate cellular and architectural atypia, which has not yet breached the basement membrane and has not invaded deeper tissue layers. The long-term clinical outcomes of LGIN are twofold (6): one is regression with the disappearance of atypia, and the other is progression to HGIN or even gastric cancer. However, there is currently no unified standard for the optimal interval of endoscopic examination, and whether immediate endoscopic resection is necessary remains controversial.

The most common method for diagnosing LGIN is endoscopic forceps biopsy (EFB). However, its limitations include a significant rate of pathological upgrade to HGIN or cancer upon resection. Studies have shown that the overall discrepancy rate between EFB and Endoscopic Submucosal Dissection (ESD) ranges from 20.1 to 76.3% (8, 9), with an eventual pathological upgrade rate of 16.3–59.4% (10–18). This uncertainty increases the complexity of clinical decision-making.

In population-based screening cohorts, the reported upgrading rates of gastric low-grade intraepithelial neoplasia are generally 3–8% (19), which are considerably lower than the rate observed in the present study. The relatively higher upgrading rate in our study may be attributed to the fact that our cohort comprised clinically referred patients who underwent endoscopic submucosal dissection, representing a higher-risk population rather than an unselected community-based screening population. This contextualization helps clarify the generalizability of the nomogram, indicating its clinical utility for patients at intermediate-to-high risk of pathological upgrading.

To improve the diagnostic accuracy of LGIN and subsequent follow-up or treatment selection, clinicians are actively exploring and trying various new technologies and methods. Among them, magnifying endoscopy with narrow-band imaging (ME-NBI) holds an important position in the clinical diagnosis of LGIN and is an important tool for clinicians (20). These new technologies enhance the resolution and contrast of endoscopic images, helping doctors more clearly observe the subtle structural changes in the gastric mucosa, thereby more accurately identifying and differentiating lesions and reducing unnecessary biopsies. Nomograms are widely used statistical prediction models in the medical field (21), especially in oncology, integrating multiple factors to assess individual patient risks or outcomes. As a predictive tool, nomograms have been applied to various tumors, achieving precise predictions of survival rates, recurrence rates, disease progression probabilities, or treatment responses. This study aims to combine pre-ESD laboratory indicators in patients to evaluate the upgrade diagnosis rate (UDR) and risk factors in EFB-confirmed gastric LGIN lesions after ESD. Based on these findings, a nomogram was developed and externally validated using data from another cohort to further demonstrate the reliability of our results. We hope to provide more accurate references for clinical diagnosis and treatment. Through this study, we aim to further optimize the management strategy for gastric LGIN, improve the accuracy of identifying patients who require early active endoscopic intervention, and thereby improve patient prognosis and quality of life.

## Materials and methods

### Patients

A retrospective analysis was conducted on clinical data from patients who underwent gastric ESD at Zhejiang Provincial Hospital of Chinese Medicine, Zhejiang Provincial People’s Hospital, and Shaoxing People’s Hospital from June 2018 to July 2024. Patients from the first two hospitals constituted the training cohort; the last hospital formed the validation cohort. The pathological upgrade group and the pathological non-upgrade group were respectively screened out from the three hospitals. Pathological upgrade group is defined as biopsy pathological results suggesting LGIN, and ESD postoperative pathological results suggesting HGIN or early gastric cancer. The pathological non-upgrade group was defined as one where both biopsy and ESD pathologies indicated LGIN.

All patients included in this study underwent ESD within 3 months after being diagnosed with LGIN by biopsy, and no HP eradication therapy was performed during this period.

The retrospective study was approved by the institutional review boards of the three hospitals (approval numbers: Zhejiang Provincial Hospital of Chinese Medicine: 2025-KLS-043-01; Zhejiang Provincial People’s Hospital: QT2025072; Shaoxing People’s Hospital: 2023-Research081-01). Considering the retrospective nature of the study and the use of de-identified patient data, the requirement for informed consent was waived. The study was conducted in accordance with the ethical standards of the Helsinki Declaration and its later amendments.

### Inclusion and exclusion criteria

Inclusion criteria: 1. Patients who underwent gastric ESD at the three hospitals and were diagnosed with early gastric cancer or HGIN were selected. The medical history of these patients was reviewed, and those with biopsy-proven LGIN at the same lesion site were included. 2. All biopsy and surgical specimens were diagnosed according to the WHO classification criteria for tumors of the digestive system. The pathological results were reviewed and confirmed by pathologists with the title of associate chief physician or above. 3. Patients with complete clinical records and endoscopic data were included.

Exclusion criteria: 1. Patients whose post-ESD pathology indicated signet ring cell carcinoma, mucinous adenocarcinoma, neuroendocrine tumor, inflammation, stromal tumor, leiomyoma, polyp, ectopic pancreatic tissue, or lipoma were excluded. 2. Patients with a history of gastric cancer or prior subtotal gastrectomy were excluded. 3. Patients whose polyps could not be clearly described in terms of shape, location, number, or size were excluded.

### Clinical data

General clinical information of patients was collected, including sex, age, smoking history, alcohol consumption history, presence of atrophic gastritis, H. pylori infection, pepsinogen, serum gastrin, and tumor markers (CEA, CA199, CA72-4).

Factors Related to Endoscopy: Professional title of the endoscopist.

Lesion-Related Data: Lesion size, location, morphology, Gastric mucosal congestion (presence of erythema or redness), Surface characteristics (whether rough, ulcerated, or eroded), Demarcation line (DL), Microsurface structure (MS), Microvascular pattern (MV).

The size of the lesion was defined by its maximal diameter and was incorporated into the study. The stomach was anatomically divided into five regions: antrum, body, fundus, angularis, and cardia. The Paris Classification was utilized to categorize macroscopically visible neoplastic lesions into three types: elevated (0-I, 0-IIa), flat (0-IIb), and depressed (0-IIc, 0-III). Surface erythema or redness was defined as a significantly redder appearance of the lesion mucosa compared to the surrounding mucosa. Surface roughness was characterized by nodular or irregularly elevated mucosa. Irregular microvascular pattern (IMVP) refers to the observation of irregular vascular textures within gastric lesions during endoscopy, featuring abnormal dilation of vessels, significant variation in vessel diameter, and peculiar vessel shapes (e.g., serpiginous curvature). Irregular microsurface pattern (IMSP) denotes the observation of irregular fine structures on the surface of gastric lesions during endoscopy, manifested by differences in glandular density and inconsistent shapes and sizes of glandular pits. DL + refers to the clear structural boundary line that appears between the cancerous area and the surrounding non-cancerous mucosa under ME-NBI. This boundary line stems from the abnormal changes in MV and MS caused by cancer tissues, which form a significant contrast with the regular structure of normal tissues. Mix-IIc is defined as a lesion morphology of IIc + IIa or IIc + IIb according to the Paris classification.

### Statistical methods

Statistical analysis was performed using SPSS version 27 and R version 4.0.1. In the training cohort, binary logistic regression analysis was employed to identify potential factors associated with pathological upgrading. Multivariate logistic regression analysis was subsequently conducted to assess these potential factors. Independent risk factors were incorporated into the R software (version 4.0.1). The RMS package was utilized to construct a nomogram for predicting the risk of pathological upgrading in LGIN. External validation of the nomogram model was carried out using the validation dataset. Receiver operating characteristic (ROC) curve analysis, based on bootstrapping, was performed to evaluate the nomogram. The discrimination of the nomogram was assessed using the area under curve (AUC), sensitivity, and specificity. Calibration curves were used to evaluate the reliability of the nomogram. Decision curve analysis (DCA) was employed to assess the clinical utility of the nomogram by evaluating net benefits across different threshold probabilities. In all analyses, a *p*-value of less than 0.05 was considered statistically significant.

## Results

**FIGURE 1:**
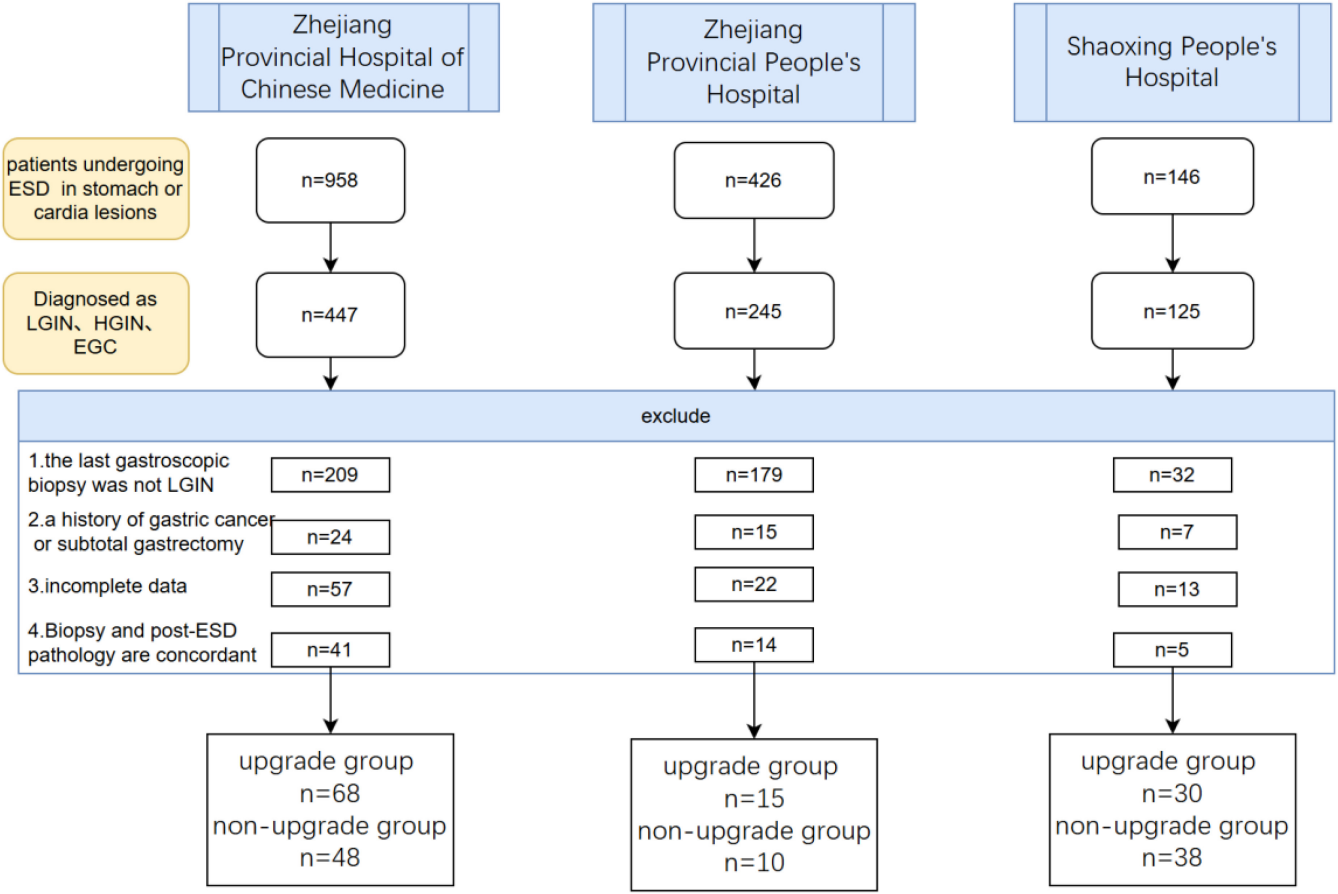
Flowchart of patient selection at three hospitals.

A total of 958 patients who underwent gastric ESD at the Zhejiang Provincial Hospital of Chinese Medicine were identified, among whom 447 were diagnosed with LGIN,HGIN or early gastric cancer. After reviewing the medical histories and applying the exclusion criteria, 68 patients were ultimately included in the pathological upgrade group, 48 patients were included in the pathological non-upgrade group, As shown in Figure 1. As shown in Figure 1, a total of 426 patients underwent gastric ESD at Zhejiang Provincial People’s Hospital. By comparing with biopsy pathology, 15 patients were included in the pathological upgrade group and 10 patients were included in the non-upgrade group.

The prediction model was established with a total of 141 patients in the training cohort, including *n* = 83 in the pathological upgrade group and n = 58 in the pathological non-upgrade group. The validation cohort consisted of 68 patients from Shaoxing People’s Hospital, including 30 in the upgraded group and 38 in the non-upgraded group.

The pathological upgrade rate in three hospital was 54.1%.

In the present study, the histological diagnoses were mainly based on the WHO classification system. To further improve the scientific rigor and readability of the manuscript, we have added representative pathological images demonstrating the progression from LGIN (biopsy) to HGIN (post-ESD) in the same cases. Details of these images are shown in Supplementary Figures 1a,b.

ROC curve analysis was performed to validate the age cutoff. The optimal cutoff identified by the maximum Youden index was 60.5 years. Given that age is a continuous variable and 60 years is a clinically meaningful and commonly used threshold in the field, we selected 60 years as the cutoff point for dichotomization in our study. This confirms that the age cutoff was statistically justified (Supplementary Figure 2).

### Clinical baseline characteristics of patients with pathological upgrade

A total of 68 patients were ultimately included from Zhejiang Provincial Hospital of Chinese Medicine, and 15 patients from Zhejiang Provincial People’s Hospital. We designated 60 years of age as the cutoff for patient age and utilized 15 mm as the thresholds for lesion size categorization. Lesion locations were classified into five distinct regions: the antrum, body, angular portion, fundus, and cardia of the stomach. Additionally, lesion morphology was simplistically divided into the presence or absence of type IIc features. Table 1 provides a detailed comparison of 13 baseline characteristics between the two cohorts and the results of their univariate logistic regression analysis.

**TABLE 1:** Clinical baseline characteristics and the outcomes of the binary logistic regression analysis of patients with pathological upgrade.

Variable		Zhejiang Provincial Hospital of Chinese Medicine (*n* = 68)	Zhejiang Provincial People’s Hospital (*n* = 15)	*P*-value	Odds ratios (OR)	95% CI
Age	<60 ≥ 60*	16 (23.5%) 52 (76.5%)	1 (6.7%) 14 (93.3%)	0.123	0.534	0.241–1.184
Sex				0.024	0.426	0.204–0.892
	Male	39 (57.4%)	13 (86.7%)			
	Female*	29 (42.6%)	2 (13.3%)			
Smoking				0.107	2.437	0.936–6.349
	Yes	13(17.4%)	9(60.0%)			
	No*	55(82.6%)	6(40.0%)			
Drinking				0.139	2.170	0.778–6.053
	Yes	10(14.7%)	5(33.3%)			
	No*	58(85.3%)	10(66.7%)			
Atrophic				0.533	1.377	0.381–4.979
	Yes	46(68.1%)	9(60.0%)			
	No	22(31.9%)	6(40.0%)			
H. pylori				0.087	1.865	0.734–1.697
	yes	14(20.6%)	3(20.0%)			
	no	54(79.4%)	12(80.0%)			
Lesion location				0.805	0.736	0.393–1.375
	Antrum	54(79.4%)	9(60.0%)			
	Body	6(8.8%)	5(33.3%)			
	Fundus	4(5.9%)	1(6.7%)			
	Angularis	2(2.9%)	0(0.0%)			
	Cardia	2(2.9%)	0(0.0%)			
Lesion size				0.021	0.372	0.161–0.863
	<15 mm*	32(46.4%)	3(20.0%)			
	>15 mm	36(53.6%)	12(80.0%)			
Morphology				0.001	5.850	2.028–16.876
	Include IIc	49(72.1%)	10(66.7%)			
	Exclude IIc*	19(27.9%)	5(33.3%)			
Surface	Erythema	46(67.6%)	11(73.3%)	0872	1.067	0.486–2.343
	Rough	33(48.5%)	2 (13.3%)	0.145	1.787	0.818–3.907
	Ulcerated or eroded	16(23.5%)	1 (6.7%)	0.417	0.704	0.301–1.645
NBI	DL +	59(86.8%)	13(86.7%)	0.000	8.857	3.259–24.070
	IMVP +	43(63.2%)	7 (46.7%)	0.618	1.214	0.578–2.550
	IMSP +	53(77.9%)	7 (46.7%)	0.969	0.931	0.297–2.920
Professional title of doctor	Attending- Physician	8(11.8%)	0			
	Associate-Chief-Physician	12(17.6%)	3(20%)	0.225	0.612	0.154–2.440
	Chief-Physician*	48(70.6%)	12(80%)	0.925	1.061	5230.310–3.627
Serological indicators	Serum gastrin	45/68	–	0.192	0.496	0.069–3.569
	PG I	45/68	–	0.881	1.361	0.111–16.655
	PG II	45/68	–	0.967		
	PG I/II	45/68	–	0.299	2.602	0.012–3.569
	Hp antibody	45/68	–	0.014	6.562	0.003 – 0.520
	CEA	45/68	–	0.634	2.326	0.072 – 75.269
	CA199	45/68	–	0.999	1.369	0.586 – 1.878
	CA724	45/68	–	0.184	8.754	0.357 – 214.675

*The reference category.

### Univariate analysis of risk factors for pathological upgrading

In the binary logistic regression analysis, five factors exhibited a *P*-value less than 0.05, indicating statistical significance. These factors include sex [Odds Ratio (OR): 0.426, 95% Confidence Interval (CI): 0.204–0.892, *P* = 0.024], lesion morphology characterized by the presence of mixed type IIc lesions (OR: 5.85, 95% CI: 2.028–16.876, *P* = 0.001), clear lesion margins (OR: 8.857, 95% CI: 3.259–24.07, *P* < 0.001), lesion size (OR: 0.372, 95% CI: 0.161–0.863, *P* = 0.021), and positivity for Hp antibodies (OR: 6.562, 95% CI: 1.612–26.718, *P* = 0.014).

Consequently, we identified male sex, mixed type IIc lesion morphology, distinct lesion margins, lesion size of 15 mm or greater, and positive Hp antibody status as independent risk factors for pathological upgrading, which were subsequently incorporated into the multivariate logistic analysis. The results of binary logistics regression analysis are shown in Table 1.

A total of 77 patients had valid H. pylori antibody data (Zhejiang Provincial Hospital of Chinese Medicine: *n* = 45 in the pathological upgrade group and *n* = 32 in the pathological non-upgrade group), corresponding to a missing rate of 45.39% (64/141) in the analytical cohort. Multiple imputation was employed for sensitivity analysis to assess the impact of missing values on the results. In sensitivity analysis, Hp antibody was not significantly associated with the outcome in either univariate (*P* = 0.218) or multivariable analysis (*P* = 0.192). Therefore, it was excluded from the final multivariable model.

### Multivariate analysis of pathological upgrading risk

A multivariate analysis was conducted on the risk factors identified from the aforementioned binary logistic regression analysis, which included lesion morphology, margin clarity, and size. The results indicated that lesion morphology classified as mixed type IIc, clear demarcation lines, and sizes ≥ 15mm are risk factors for pathological upgrading. Please refer to Table 2 for details.

**TABLE 2:** Multivariate analysis of pathological upgrading risk.

Risk factors	OR	95%CI	*P*
Sex	0.675	0.279–1.633	0.384
Lesion morphology (mixed IIc)	7.819	2.252–27.152	0.001
Lesion size	0.229	0.078–0.666	0.007
Lesion DL +	8.940	2.867–27.874	<0.001

### Construction of a nomogram for predicting the risk of pathological upgrading

**FIGURE 2:**
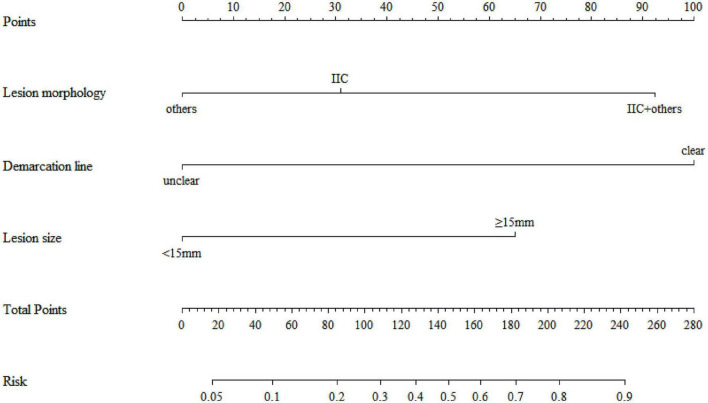
A nomogram for predicting the risk of pathological upgrading.

Based on the outcomes of logistic regression analysis, we utilized the RMS package in R software to construct a nomogram, as depicted in Figure 2. For lesions with morphology type IIc, the corresponding score is 31 points; when the lesion morphology is a mixed type IIc focus, the score reaches up to 90 points; for lesion sizes ≥ 15 mm, the score is 65 points; and a clear boundary also scores over 90 points. Lesions fulfilling all risk factors warrant strong recommendation for endoscopic intervention. For those exhibiting 1–2 risk factors, endoscopic management or repeat biopsy within a defined timeframe is advised. In the absence of these risk factors, extended surveillance intervals may be considered.

The training cohort included 141 patients, among whom 83 patients experienced pathological upgrading. A total of 4 predictors were incorporated into the multivariable prediction model. The number of events per variable (EPV) was calculated as 83/4 = 20.75, which clearly met the widely accepted 10 EPV rule and the sample size criteria for prediction model development proposed by Riley et al. (22).

### ROC curves

**FIGURE 3:**
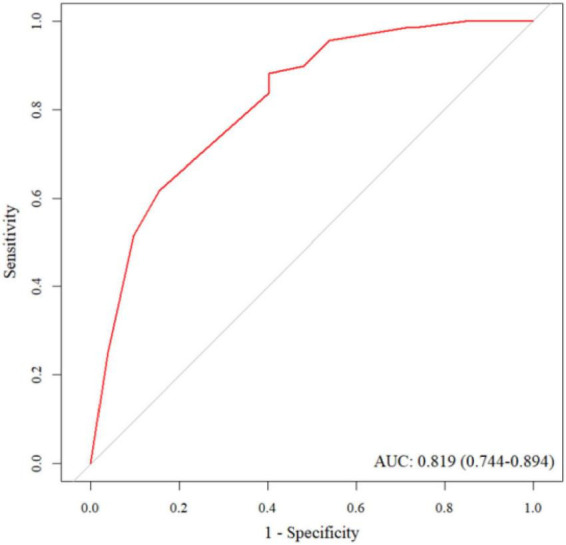
The ROC curve for the training set.

**FIGURE 4:**
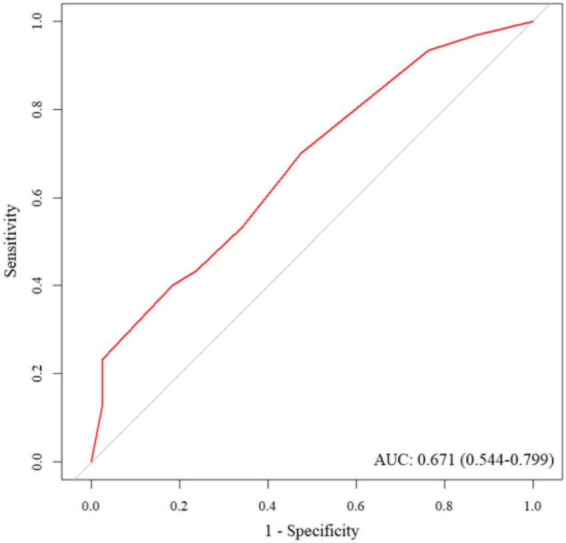
The ROC curve for the validation set.

The AUC for the training set was 0.819, indicating a satisfactory level of discriminative ability. The sensitivity and specificity of the training set were 0.882 and 0.596, respectively. The AUC for the validation set was 0.671, suggesting moderate discriminative power. The sensitivity and specificity for the validation set were 0.700 and 0.526, respectively. The ROC curve for the training set is illustrated in Figure 3, while the ROC curve for the validation set is shown in Figure 4.

Furthermore, we performed additional ROC curve analyses to evaluate changes in model performance. After including sex and Hp antibody status in the model, the AUC of the training cohort model decreased from 0.819 to a range of 0.569–0.701, indicating a significant reduction in discriminative ability.

### Clinical decision-making curve analysis

**FIGURE 5:**
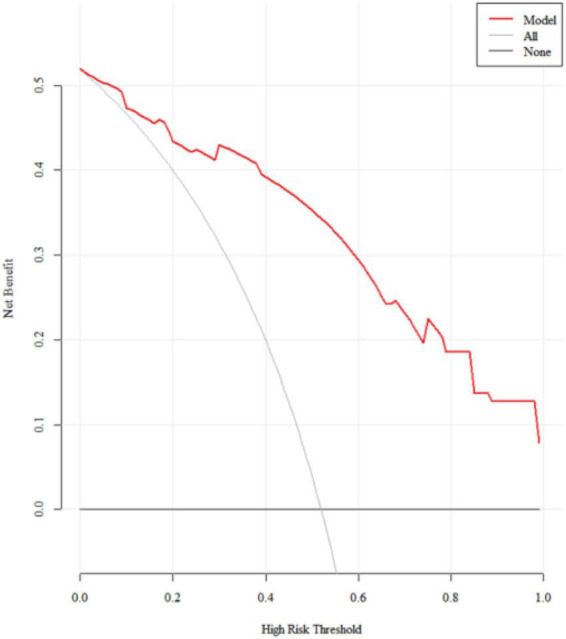
The DCA for the training set

**FIGURE 6:**
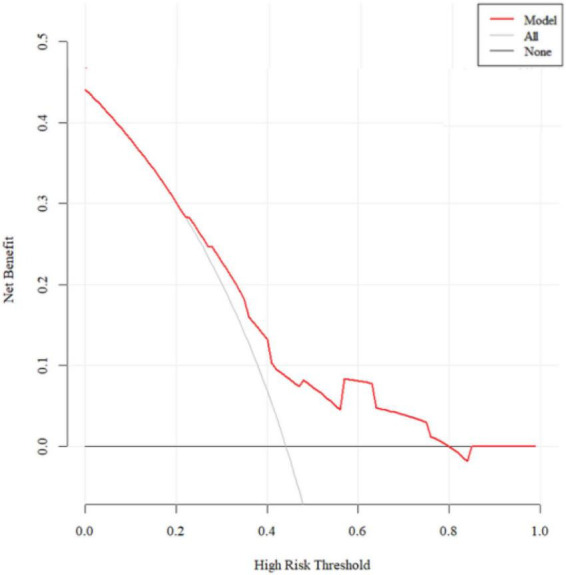
The DCA for the validation set.

The decision curve analysis (DCA) for the training set is depicted in Figure 5. The model demonstrates a net benefit that surpasses both “intervention for all” and “no intervention” across nearly all threshold probabilities, indicating a substantial clinical benefit. The DCA for the validation set is shown in Figure 6, where the model’s net benefit is higher than both “intervention for all” and “no intervention” within the threshold range of 23–79%, signifying considerable clinical utility.

### Calibration curves

**FIGURE 7:**
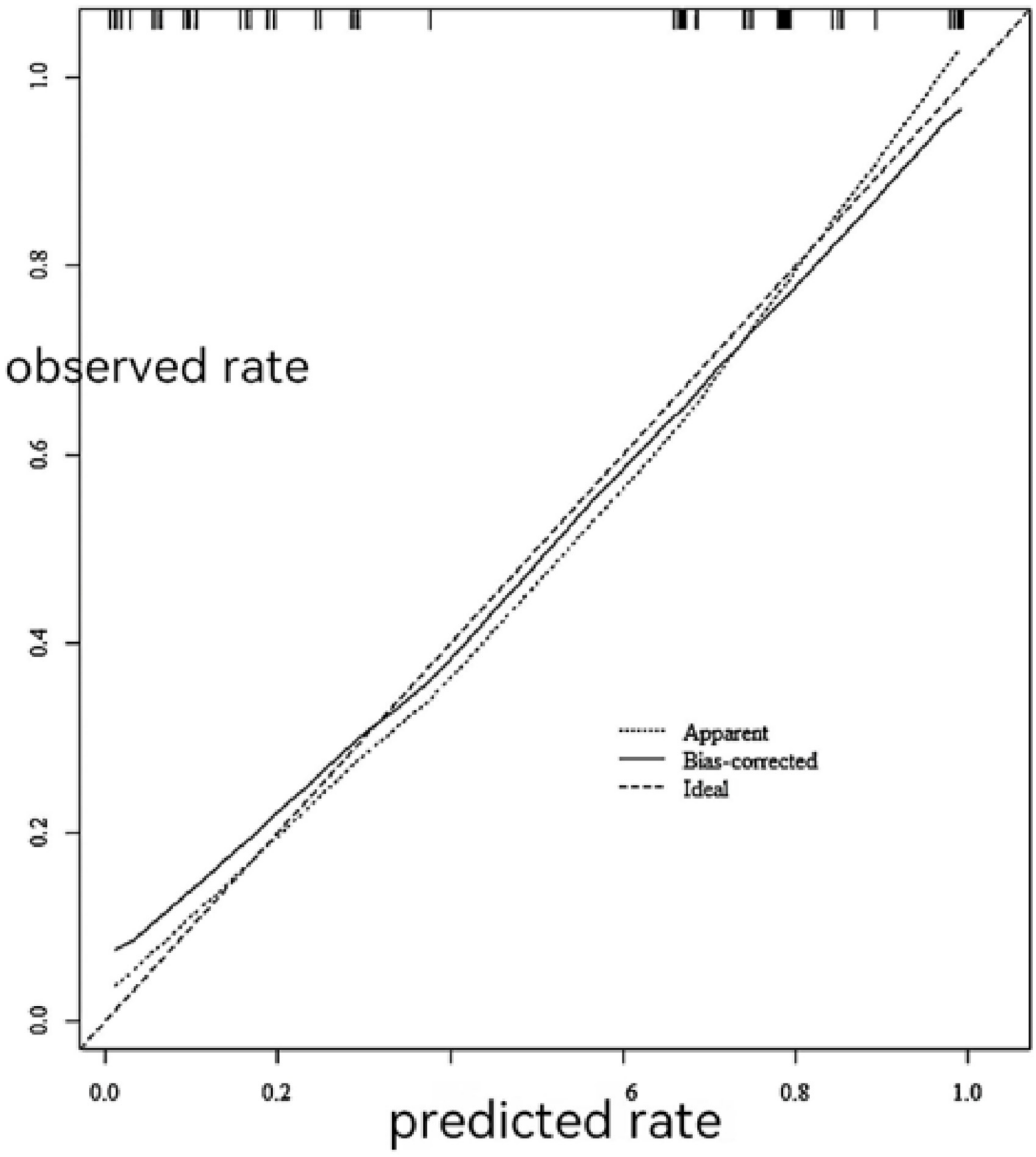
The calibration for the curve for the training set.

**FIGURE 8:**
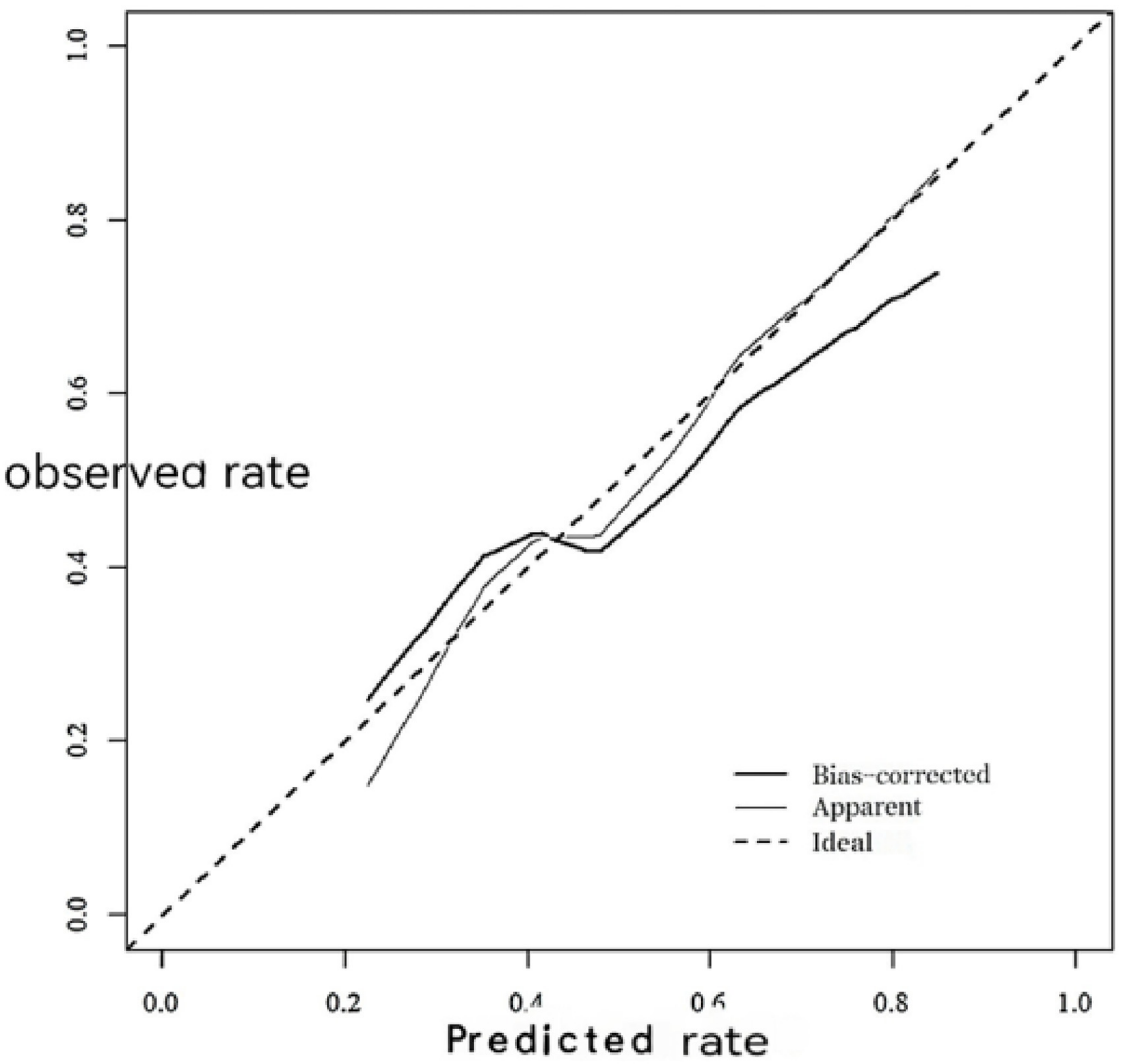
The calibration for the curve for the validation set.

The calibration curve for the training set is presented in Figure 7, brier score: 0.038. The calibration curve for the validation set is shown in Figure 8, brier score: 0.041. The proximity of the calibration curves for both cohorts to the diagonal line indicates that the predictive model possesses satisfactory calibration ability.

The validation cohort’s calibration curve seems to deviate from the diagonal in certain probability ranges. Potential causes include: small validation sample size (*n* = 68), sample heterogeneity, and unmeasured confounders. In future retrospective analytical studies, we will further expand the sample size to improve the reliability and generalizability of the research results.

## Discussion

Our study identified male sex, lesion size of 15 mm or larger, clear demarcation line, mixed lesion morphology, and positive Helicobacter pylori antibody status as risk factors for the pathological progression of LGIN. Upon further multivariate analysis, sex and Hp antibody status were excluded as independent risk factors. Although our study did not conclusively establish male sex as a risk factor, in the study by Chen et al. (23), male sex was incorporated as a risk factor in the nomogram analysis, which aligns with the higher incidence of gastric cancer in males (24). The risk of pathological progression in male patients may interact with factors such as lesion diameter, morphology, and the presence of erosions or ulcers; the specific mechanisms of action require further investigation for validation.

At present, there is no consensus among experts regarding the specific size of lesions as a risk factor for pathological upgrading. In our study, we chose 10, 15, and 20 mm as the cutoff points based on previous relevant literature and commonly used clinical thresholds in this field. A cutoff of 15 mm was statistically significant (*P* = 0.021; OR = 0.372, 95% CI: 0.161–0.863). A cutoff of 10 mm showed a non-significant trend (*P* = 0.097; OR = 0.448, 95% CI: 0.174–1.155). A cutoff of 20 mm also showed a borderline non-significant trend (*P* = 0.051; OR = 0.405, 95% CI: 0.163–1.004). Given the statistical significance and clinical relevance, we selected 15 mm as the primary cutoff value for lesion size in our analyses, which is in line with the views of Won et al. (25). However, Ryu et al. (26) have identified lesions ≥ 10 mm (OR:2.200; 95% CI:1.393–3.474) as a risk factor, while Lim H et al. (27) designated endoscopic features of lesions with a maximum diameter ≥ 18 mm as risk factors. A 2015 meta-analysis by Zhao et al. (14) mentioned that lesions ≥ 20 mm are significant risk factors for diagnostic upgrading following endoscopic treatment, although this meta-analysis had high heterogeneity, included a limited number of studies, and was conducted early, potentially influenced by endoscopic factors. Therefore, our paper still opts for multi-threshold analysis of lesion size and ultimately selects a cutoff value of 15 mm for investigation.

As early as 2016, Muto et al. introduced a straightforward diagnostic algorithm for early gastric cancer under ME-NBI, which encompasses morphology, arrangement distribution, and atypia (28). Performing NBI examinations provides substantial diagnostic value in assessing whether patients with biopsy- confirmed gastric LGIN should undergo ESD and whether the pathology will be upgraded post-ESD. Although our findings only suggest that DL positive under NBI is a risk factor, a retrospective review of the data reveals that among patients with pathological upgrades, the presence of IMVP + or IMSP + exceeds 50%. Patients who are positive for all three—DL, IMVP, and IMSP—account for 32 out of 68 (47%), and those with DL + in conjunction with IMVP + or IMSP + are as high as 64 out of 68 (94.1%). This aligns with the simplified algorithm proposed by Muto et al. and also illustrates the crucial role that NBI plays in lesion assessment and enhancing physicians’ understanding (29, 30).

Previous literature predominantly identifies depressed lesions as risk factors for pathological progression (12–15, 25, 27, 31), aligning with our conclusions. However, the findings of this paper provide a more detailed perspective, suggesting that lesions with a mixed pattern including type IIc are more likely to be associated with an increased risk of pathological upgrading. Depressed lesions are often associated with inflammatory responses and microvascular abnormalities, which may indicate that the lesions have involved the submucosal layer or deeper tissues of the stomach, potentially leading to insufficient sampling depth during endoscopic biopsy and an underestimation of the severity of the lesions. Mixed lesions pose greater concern as they may encompass elements of LGIN, HGIN, or EGC. This heterogeneity increases the potential risk of lesion progression, thereby raising the likelihood of pathological upgrading.

Given the high missingness of HP serological antibody status (available in 77 of 141 patients), and its non-significant association with pathological upgrading in sensitivity analyses, Hp antibody positivity was excluded from the final multivariable model. A review of the data revealed that among patients with positive Hp antibodies, the probability of pathological upgrading reached 15 out of 19 cases (78.9%). Literature indicates that serum positivity for Hp antibodies, along with low expression of PG I and high expression of PG II, are influential factors for precancerous lesions of gastric cancer (OR > 1, *P* < 0.05). Hp antibodies reflect the status of Hp infection over a period and are not affected by recent medication use or local gastric lesions. Although Hp antibodies are not typically used as a primary diagnostic tool in clinical practice, a positive Hp antibody suggests past or present infection, which could potentially damage the gastric mucosa and increase the risk of pathological progression, even if Hp has been eradicated. For patients with positive Hp antibodies, especially those who are also positive for Hp, it is crucial to closely monitor and manage gastric mucosal lesions, and it is recommended to determine the nature of the lesions as soon as possible.

Several potential factors contribute to the risk of pathological upgrading: 1. Inaccuracy in sampling during endoscopic biopsy (32). For instance, when lesions are small and only exhibit focal precancerous changes, they may be challenging to detect via endoscopic biopsy. Furthermore, some lesions are located deeper, where endoscopic biopsies typically sample only the mucosal epithelial and lamina propria layers, making it difficult to access deeper lesions. Therefore, it is recommended to clearly identify the tumor area under endoscopy, avoid necrotic parts, and take multiple samples to increase the positive biopsy rate. 2. The location of the lesion can also affect the detection rate of early cancer (26). Early gastric cancers in the cardia or body of the stomach, especially on the lesser curvature or posterior wall, are often overlooked. although our study did not find an impact from lesion location. 3. Endoscopist experience and pathologist interpretation subjectivity (5). In our study, the experience of endoscopists was not included as a risk factor, possibly due to societal factors lead patients to prefer treatment by more experienced senior physicians. Further research should consider incorporating more detailed data on physician experience, such as the number of years performing endoscopy and endoscopic training history.

The strengths of our study are as follows. First, we incorporated patient data from three geographically distinct top-tier hospitals, which further substantiates the reliability of our findings. Second, our study evaluated a broad range of factors, and for the first time, we identified Hp antibody status and mixed lesion morphology as risk factors for pathological progression in univariate analysis, particularly lesions with type IIc components, which scored over 90 points on the nomogram, indicating a higher level of risk.

This study has several limitations. Firstly, the retrospective design may lead to selection bias among the patients, particularly regarding the timing of ESD and the history of H. pylori eradication; secondly, the sample size was relatively small, this small sample size led to excessively low statistical power; thirdly, the study included patients undergoing ESD and excluded other Endoscopic Resection (ER) techniques like EMR, although many researchers advocate the use of ER for gastric LGIN (33). Fourthly, only 45 patients completed all serological indicators, the small sample size due to missing data may limit the statistical power and generalizability of the findings related to HP serological status. Future prospective studies are needed to confirm the role of serological markers. Consequently, the conclusions have limitations. In the future, the authors plan to expand research facilities and increase the number of patients to enhance the representativeness of the data.

## Conclusion

This multi-center retrospective study determined a pathological upgrade rate of 54.1% for gastric LGIN. It also identified several independent risk factors associated with the progression of LGIN to HGIN or gastric cancer. These factors encompass lesion morphology that is mixed, clear demarcation line, and lesions ≥ 15 mm in size. Although the results of the validation cohort of the nomogram indicate that its predictive performance is moderate, we cannot deny its clinical value in improving clinical management and decision-making for this precancerous condition. Future prospective studies are essential to further substantiate the nomogram’s performance across diverse clinical settings.

## Data Availability

The datasets presented in this article are not readily available because. The data in this study cannot be shared due to ethical restrictions and participant privacy protection. Requests to access the datasets should be directed to Di-Yun Shen, shendiyun456101@163.com.
