# Supplementary material for: A multicenter retrospective study: risk factors and nomogram construction for pathological escalation of gastric low-grade intraepithelial neoplasia
Source: Front Med (Lausanne). 2026 Apr 30;13:1800141. doi: 10.3389/fmed.2026.1800141 (PMC13171808; doi:10.3389/fmed.2026.1800141)

A multicenter retrospective study: Risk factors and nomogram construction for pathological escalation of gastric low-grade intraepithelial neoplasia

AUTHOR: Di-yun Shen^1^, Shun-hai Zhou^2^, Chao-yi Shi^2^, Xuan-ran Chen^3^, Yu-zhen Bi^4^, Xue-man Wang^5^, Yan-Sun^6^, Jun Zhang^7^*

| 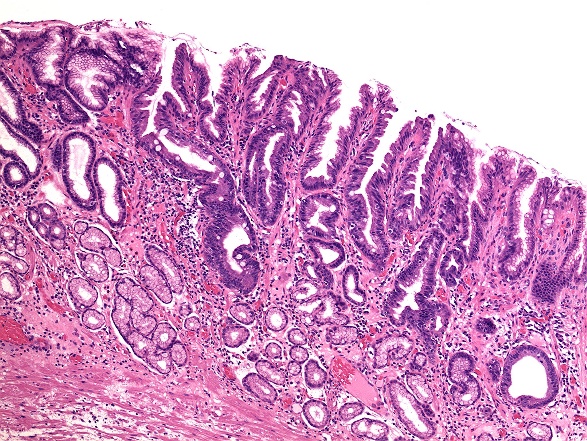Supplementary Figure1a: LGIN (100*magnification) | 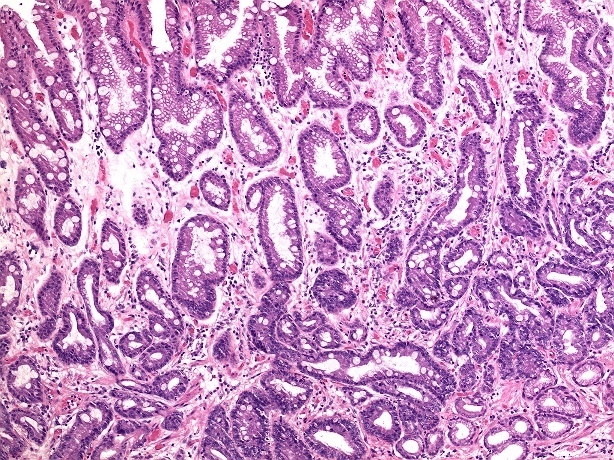Supplementary Figure1b: HGIN (100*magnification) |
| --- | --- |
|  |  |


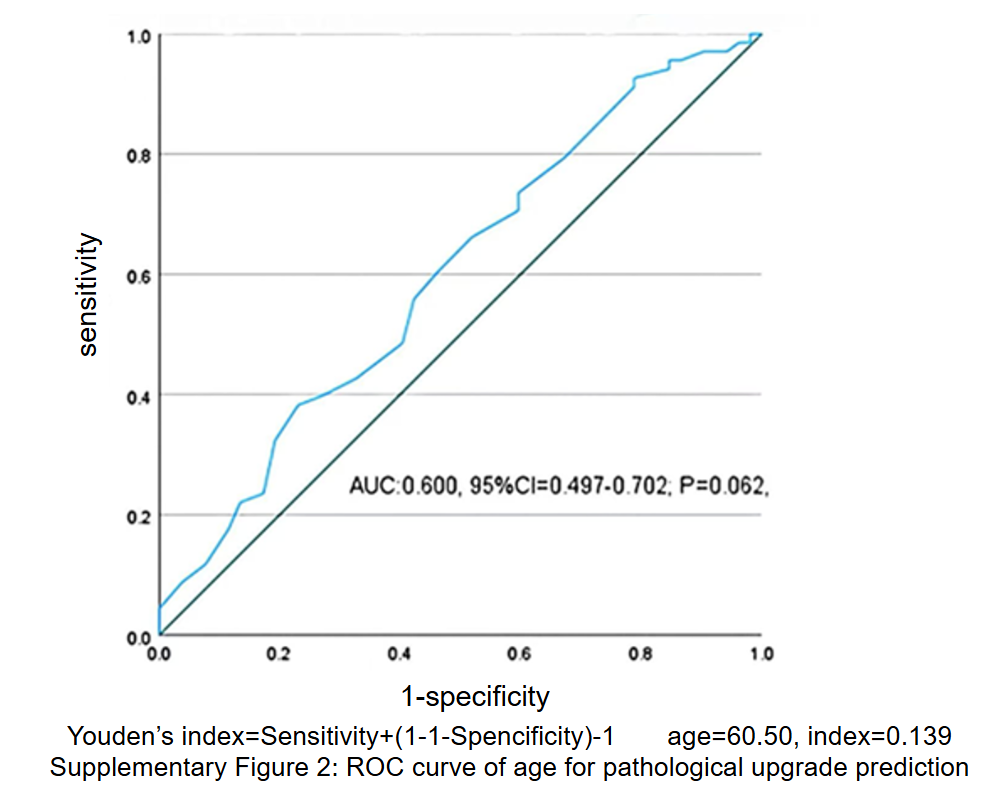

Supplement: Supplementary file 1 [file Supplementary_file_1.docx]
